# Rhodium-Based Catalysts: An Impact of the Support Nature on the Catalytic Cyclohexane Ring Opening

**DOI:** 10.3390/nano13050936

**Published:** 2023-03-04

**Authors:** Kristina E. Kartavova, Mikhail Yu. Mashkin, Mikhail Yu. Kostin, Elena D. Finashina, Konstantin B. Kalmykov, Gennady I. Kapustin, Petr V. Pribytkov, Olga P. Tkachenko, Igor V. Mishin, Leonid M. Kustov, Alexander L. Kustov

**Affiliations:** 1 Department of Chemistry, Lomonosov Moscow State University, 119991 Moscow, Russia; 2 Institute of Ecotechnologies, National University of Science and Technology “MISiS”, 119049 Moscow, Russia; 3 N.D. Zelinsky Institute of Organic Chemistry RAS, 119991 Moscow, Russia

**Keywords:** rhodium, support, cyclohexane, ring opening, heterogeneous catalysis, gas phase reaction, cetane number, fuel upgrading

## Abstract

Because of the growing demand for high-quality fuels, the light cycle oil fraction improvement including cetane number improvement is important. The main way to reach this improvement is the ring opening of cyclic hydrocarbons, and a highly effective catalyst should be found. Cyclohexane ring openings are a possible option to investigate the catalyst activity. In this work, we investigated rhodium-loaded catalysts prepared using the commercially available industrial supports: single-component ones, SiO_2_ and Al_2_O_3_; and mixed oxides CaO + MgO + Al_2_O_3_ and Na_2_O + SiO_2_ + Al_2_O_3_. The catalysts were prepared by incipient wetness impregnation and investigated by N_2_ low-temperature adsorption-desorption, XRD, XPS, DRS UV-Vis and DRIFT spectroscopy, SEM, and TEM with EDX. The catalytic tests were performed in cyclohexane ring opening in the range of 275–325 °C. The best result was demonstrated by the sample 1Rh/CaMgAlO: the selectivity to *n*-hexane was about 75% while the cyclohexane conversion was about 25% at 275 °C. The space-time yield was up to 12 mmol*_n_*_-hexane_ g_cat_^−1^h^−1^.

## 1. Introduction

High-quality fuels nowadays are highly demanded and the demand is steadily and significantly increasing. There is an option to blend the diesel fuel with the light cycle oil (LCO) fraction. Such an option is possible because the composition of this fraction is appropriate in terms of the number of carbon atoms. However, there is a problem to meet the standards of diesel fuel in the cetane number (the desired cetane number is ≥47) and the polyaromatics content. Hydrogenation of polyaromatics can partially solve the problem and decrease the content of polyaromatics, but the production of naphthenic substances does not solve the problem of the cetane number, so the ring-opening process should be applied [1]. The comparison of the cetane number of different hydrocarbons demonstrates that the linear alkanes are the most preferable ones, and much less preferable but still appropriate are the monobranched alkanes. At the same time, the length of the carbon chain plays a significant role: the longer the chain, the higher the cetane number [2,3]. That is why the highly active and selective linear alkanes catalysts of the ring-opening reaction should be found to improve the cetane number of the diesel produced from LCO.

In order to investigate the process of ring opening, different cyclic hydrocarbons are used: mostly decalin, but also tetralin, perhydroindane, methylcyclohexane, etc. [4]. As a model reaction of ring opening, cyclohexane opening can be applied [5,6,7], because the cyclohexane molecule is the simplest cyclic hydrocarbon with good stability. Sometimes other cyclic hydrocarbons are used [4].

The catalysts applied in the ring opening reaction can be monofunctional acidic catalysts or metal catalysts deposited on the acidic or non-acidic supports to form bifunctional or monofunctional metallic catalysts, respectively [8]. By using decalin transformations, the following mechanisms were proposed: the mechanism through carbocation formation on the monofunctional acid catalysts promoting the isomerisation; hydrogenolysis/hydrocracking mechanism over the monofunctional metallic catalysts leading to a higher ring opening selectivity and lower selectivity to branched products but in this case, the conversions of decalin are low; and the bifunctional ring-opening mechanism, which seems to be the optimal one for LCO improvement because of the rather high activity and selectivity to ring-opening products and lower selectivity to branching and hydrocracking [9].

Despite the attempts to use non-noble metal catalysts, such as W-based [1] or Ni-Mo-based [10], the most active and selective catalysts are so far the catalysts based on noble metals. From the reported supported noble metal catalysts based on Rh, Ru, and Pt, the most active and selective to linear alkanes were the Rh-based ones [7]. Ir-based catalysts are sometimes applied, they also demonstrate rather good performance in the ring opening [4,11]. Bimetallic catalysts are reported to be rather active and selective, but the comparison of them with monometallic systems not always demonstrates significant improvement: nevertheless, in some cases, the yields to the products of the ring-opening reaction are higher than for monometallic catalysts [12]. The time dependence of the catalytic performance was evaluated for the bimetallic Pt-Ir catalysts with different compositions, and it was shown that the product yield distribution changed in time: with increasing time on stream, carbon deposition occurs, while the ring contraction processes are suppressed and the ring opening conversion increases [13]. Nevertheless, Rh as an active metal is widely used and also it seems to be rather promising in the reaction of cyclohexane ring opening, so the investigation of the systems based on this metal are to be conducted.

The monometallic catalysts *x*Rh/SiO_2_-Al_2_O_3_ with different Rh loadings were investigated in the decalin ring opening and it was revealed that there was an optimal Rh content for the ring opening and suppression of any other processes, in this case, it was 1.5 wt% [14]. The thermodynamic analysis shows that the optimal temperature for a decalin ring opening is about 225 °C [15]. At the same time, an increase in the hydrogen/hydrocarbon ratio leads to increasing thermodynamic yields of the ring opening process and to decreasing dehydrogenation product yields [15].

A large number of studies are devoted to the estimation of the support nature impact on the catalytic activity: the traditional supports are usually applied such as zeolitic materials or porous alumina, silica, or titania [16,17,18]. An example of the successful application of SBA-15 silicate has been reported [19]. It is proven that both the acidic sites of the support and the metal sites play a certain role in the process [20]. It has been reported that the acidic sites of a medium strength favour the process of ring opening along with isomerisation and cracking, but the cracking process plays a significant role in the case when strong acid sites are present in the zeolite [21]. The influence of residual chlorine is investigated for Pt, Rh, and Ir catalysts, and it is revealed that the behaviour of the catalyst depends on the metal and support nature. Particularly, the Rh catalysts demonstrate both the direct cyclic hydrocarbon conversion and the bifunctional mechanism with the participation of the acidic sites of the chlorinated support [22]. The attempts to create a single catalyst that is capable of providing aromatics hydrogenation and the following ring opening reaction are described, for example [23,24].

This work is aimed at the preparation of Rh catalysts on the commercial supports: silica and alumina-based, their physicochemical characterisation and catalytic tests in the reaction of selective cyclohexane ring opening to explore the influence of the support on the catalytic activity and selectivity to certain products.

## 2. Materials and Methods

### 2.1. Materials

In this work, we used the following reagents: the supports SiO_2_ (CAS 7631-86-9), Al_2_O_3_ (CAS 1344-28-1), the mixture of CaO + MgO + Al_2_O_3_ (CAS 1305-78-8, 1309-48-4, 1344-28-1), the mixture of Na_2_O + SiO_2_ + Al_2_O_3_. All the supports were purchased from Saint Gobain (Courbevoie, France) and were fractioned to obtain the particles with a size of 0.25–0.5 mm. (NH_4_)_3_RhCl_6_·*x*H_2_O (*x* ≈ 1) from Alfa Aesar (Ward Hill, MA, USA) and distilled water were used for the catalyst preparation.

### 2.2. Methods

Textural analysis of the supports was performed by measuring N_2_ adsorption-desorption isotherms at 77 K using an ASAP 2020 Plus unit (Micromeritics, Norcross, GA, USA). The BET, BJH, and *t*-plot techniques were used to derive the specific surface area and porosity from the adsorption-desorption isotherms. The micropore size distribution was derived from the DFT model of cylindrical pores for an oxide surface. Before N_2_ adsorption, the samples were evacuated at 300 °C at 10^−5^ Torr for 4 h.

SEM images and EDX data were collected using an electron microscope LEO EVO 50 XVP (Karl Zeiss, Oberkochen, Germany) with an energy-dispersive spectrometer INCA—Energy 350 (Oxford Instruments, Abingdon, UK).

Transmission electron microscopy studies were performed using an aberration-corrected JEOL JEM-2100F/Cs (JEOL Ltd., Tokyo, Japan) equipped with EDX.

The XPS spectroscopic characterisation was performed with an X-ray photoelectron spectrometer PHI5000 Versa Probe II with the source of excitation—monochromatized Al Kα radiation (1486.6 eV, 50 W). The spot of analysis was 200 μm. The powders made from the catalysts were pressed into an In foil to make a continuous layer. Element analyses were performed by the method of the relative element sensitivity factors using integral intensities of the following lines: O 1s, Si 2s, Al 2p, Rh 3d. High-resolution spectra were collected at the transmission energy of 23.5 eV. The density of the data collection was 0.2 eV per step. Binding energies were derived from the high-resolution spectra. The binding energy scale was calibrated by the lines of Au 4f_7/2_—83.96 eV, and Cu 2p_3/2_—932.62 eV.

UV-Vis diffuse reflectance spectra were collected using a spectrophotometer Shimadzu UV-3600 Plus (Shimadzu Corp., Kyoto, Japan) equipped with an integrating sphere ISR-603 at room temperature. The range of wavelengths was 300–700 nm. Barium sulphate was used as a diluent.

DRIFT spectra were collected using a spectrometer NICOLET Protege 460 equipped with a diffuse reflectance console engineered at the Zelinsky Institute of Organic Chemistry [25] at room temperature. The range of the wavenumbers was 6000–400 cm^−1^ with a 4 cm^−1^ step. The number of the collected spectra was 500 to obtain the appropriate signal-to-noise ratio. Before the spectra collection, the samples were heated at 400 °C under the pressure of 10^−3^ Torr for 2 h, the rate of heating was 5 °C per minute. CD_3_CN was chosen as a test molecule. Adsorption of CD_3_CN was conducted at room temperature and saturated vapour pressure of 96 Torr. Intensities of the absorbance bands were derived in Kubelka-Munk (KM) units. A powder of CaF_2_ was used as a standard. Spectra collection and processing were performed with the OMNIC software (ThermoFischer Scientific Corp., Waltham, MA, USA). The spectra were presented as a difference between the spectra before and after adsorption.

TPR-H_2_ studies were carried out in a half-automatized flow system with a water trap cooled down to −100 °C. The detector (TCD) was calibrated by reduction of CuO (Aldrich-Chemie GmbH, Burlington, MA, USA, 99%). Prior to the measurements, the samples were pretreated in an Ar flow with a gas flow rate of 30 mL min^−1^ at 300 °C. Then the catalysts were heated to 850 °C at the rate of 10 °C/min in a 5% H_2_−Ar gas mixture.

X-ray diffraction analysis was performed after the calcination of the samples using an ARL X’TRA diffractometer (ThermoFischer Scientific Corp., Waltham, MA, USA) with CuKα radiation (40 kV, 40 mA) with a scanning rate of 1.2° per minute over the scanning range of 10 < 2θ < 70°. ICCD data were used for identification purposes.

The catalytic reaction of cyclohexane ring opening was performed at temperatures in the range of 275–325 °C, the pressure was 40 atm, the feed mixture consisted of 0.0170 mL of liquid cyclohexane further vaporized per min, and H_2(g)_ (50 mL per min). Therefore, the molar ratio H_2_/C_6_H_12_ was 14. The analysis of the reaction products was conducted with a gas chromatograph Chromatec Crystal 5000.2 equipped with two thermal conductivity detectors and two flame ionisation detectors, and capillary columns Hayesep 1 m × 2 mm, NaX 3 m × 2 mm, Hayesep 3 m × 2 mm, GasPro 60 m × 0.32 mm, and ZB-1 60 m × 0.32 mm. The data were processed with the software package Chromatec Analytic 3 (Chromatec Ltd., Ioshkar-Ola, Russia). The catalyst loading was 0.3 cm^3^, the masses were 100–150 mg, and the size of the particles was in the range of 0.25–0.5 mm.

The selectivity to *n*-hexane (*s*) and space-time yield (STY) of *n*-hexane were calculated by the following formulae:

$$s=\frac{n_{C_{6}H_{14}}}{n_{C_{6}H_{14}}+\sum n_{i}},$$

where *n_i_* is the number of moles of each product of the reaction excluding *n*-hexane.

$$STY=\frac{X_{C_{6}H_{12}}s\;n_{cyclohexane}^{inlet}}{m_{catalyst}},$$

where *X*(C_6_H_12_) is the cyclohexane conversion, *s* is the selectivity to *n*-hexane, *n*^inlet^_cyclohexane_ is the inlet flow of cyclohexane (mmol h^−1^), and m_catalyst_ is the mass of the catalyst (g).

### 2.3. Synthetic Procedure

The syntheses were performed by the wet impregnation method of the commercial supports: SiO_2_, Al_2_O_3_, the mixture of CaO + MgO + Al_2_O_3_, and the mixture of Na_2_O + SiO_2_ + Al_2_O_3_. The supports were impregnated with a solution prepared from (NH_4_)_3_RhCl_6_·H_2_O to obtain the catalyst with the following composition: *m*_Rh_/(*m*_Rh_ + *m*_support_) = 0.01. The impregnated samples were dried at 60–90 °C for 2 h and calcined at 550 °C for 4 h. The temperature of the calcination and the reduction were chosen on the basis of TG and TPR-H_2_ analysis of a model sample presented in Supplementary Materials (Figure S1). The obtained catalysts were denoted as 1Rh/SiO_2_, 1Rh/Al_2_O_3_, 1Rh/CaMgAlO, 1Rh/NaSiAlO. Rhodium(III) oxide was reduced with H_2_ before the catalytic tests at the fixed-bed flow-type stainless steel reactor under an H_2_ flow of 50 mL per min at 450 °C (*p*H_2_ = 40 atm) for 3 h.

## 3. Results

### 3.1. BJH–BET Measurements

**Figure 1:**
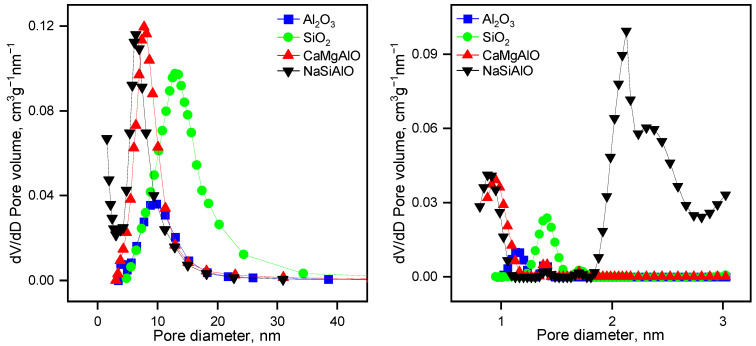
Meso- (**left**) and micro- (**right**) pore size distributions for the samples of the supports.

The supports were examined by the low-temperature N_2_ adsorption-desorption method (Figure 1, Table 1). All the samples are mostly mesoporous, with a small volume of micropores. The surface areas are rather large, and except for Al_2_O_3_, the surface areas are larger than 200 m^2^g^−1^. The mesopore size distribution maxima are different, and they increase in the following sequence: NaSiAlO < CaMgAlO < Al_2_O_3_ < SiO_2_. The total pore volume of the samples also differs, and the most porous sample is SiO_2_, whereas the least porous material is Al_2_O_3_. Pore size distributions of the mixed supports are similar to each other. The DFT model calculations revealed pore size distributions in the region of micropores. Noticeably, the mixed supports CaMgAlO and NaSiAlO have almost the same PSD in the region of micropores, but the sample NaSiAlO has an additional PSD peak at the border between micro- and mesopores. Since the peak is located in the region of small mesopores, it affects the surface area significantly. That is why the surface area of the sample NaSiAlO is the largest.

The isotherms of nitrogen adsorption-desorption are presented in Supplementary Materials (Figure S2). All the isotherms are of type IV according to the IUPAC classification [26].

### 3.2. XRD

**Figure 2:**
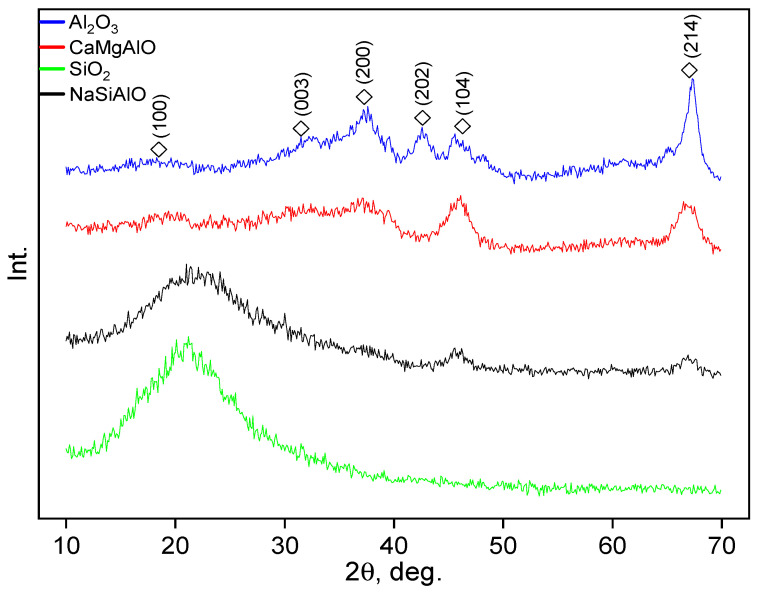
Diffractograms of the supports. The figure contains the reference reflexes of the phase γ-Al_2_O_3_ shown as M: the ICDD card number [00-013-0373].

The samples of the supports were investigated by XRD. The SiO_2_ sample demonstrates only a wide halo in the range of 10–30 degrees (Figure 2). It should be assigned to amorphous silica. The intensity of such a halo in the case of the SiO_2_ sample is larger than that in the case of NaSiAlO, seemingly such behaviour should be attributed to a different silica phase content in the sample. The mixed support NaSiAlO demonstrates a wider halo at 10–30 degrees and additional weak wide reflexes of Al_2_O_3_. The diffractograms of the supports Al_2_O_3_ and CaMgAlO demonstrate only the reflexes referred to as the -Al_2_O_3_ phase. Noticeably, the intensity of the reflexes corresponding to Al_2_O_3_ decreases in the following order: Al_2_O_3_ > CaMgAlO > NaSiAlO. This can be caused by a decrease in the Al_2_O_3_ phase amount.

### 3.3. SEM and EDX Characterisation

**Figure 3:**
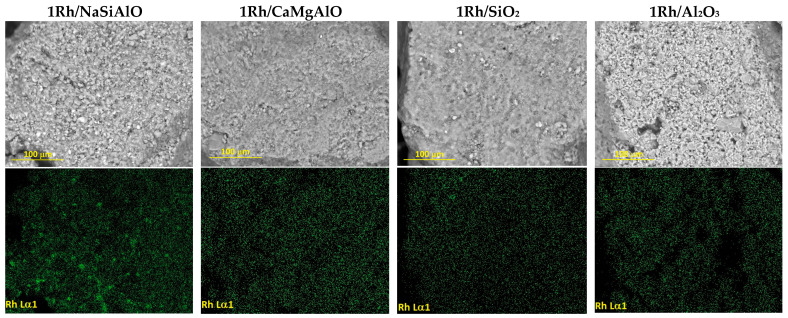
SEM images (the upper row) and rhodium distribution maps (the lower row) of the rhodium-containing catalysts.

The SEM examination of the prepared catalysts revealed the difference in the rhodium distribution on the surfaces (Figure 3). In the case of the catalysts with single-component supports, 1Rh/SiO_2_ and 1Rh/Al_2_O_3_, deposited rhodium are distributed evenly on the surfaces. The black side areas on the Rh maps and microphotos can be attributed to the areas out of the support particles.

The catalysts with mixed supports, 1Rh/NaSiAlO and 1Rh/CaMgAlO, demonstrate a difference in the rhodium distribution: the sample 1Rh/NaSiAlO has inhomogeneous rhodium distribution, i.e., the areas with higher Rh atoms concentrations on the catalyst surface can be seen as light spots on the picture, and the regions with a small Rh atoms concentration can be found as dark ones. It means that the areas of higher and lower Rh concentrations can be seen on the map.

So, all the samples, excluding 1Rh/NaSiAlO, have evenly distributed Rh on their surfaces, but the sample 1Rh/NaSiAlO has an inhomogeneous rhodium distribution.

Additional SEM images and the results of EDX can be found in Supplementary Materials, Table S1.

### 3.4. TEM

**Figure 4:**
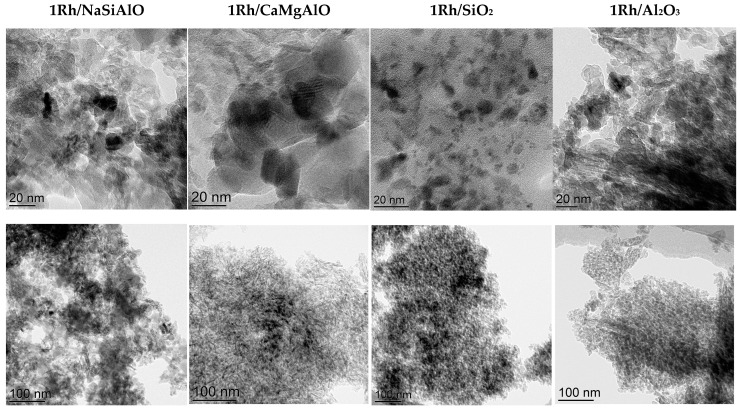
TEM images of the initially calcined in air catalysts with different supports.

All the prepared catalysts were examined by TEM (Figure 4). TEM investigation of the rhodium-loaded catalysts revealed the difference in the particle size distribution. 1Rh/Al_2_O_3_ contains observable particles with a size of about 20 nm, CaMgAlO—18–23 nm, NaSiAlO—13–19 nm, and SiO_2_—2–8 nm. So, the smallest particles are present in the SiO_2_-supported catalyst, and the largest—in the CaMgAlO-based catalyst. Rhodium is present not in all the EDX spectra for each sample. It may indicate that Rh atoms do not cover all of the surfaces, but it is distributed in a form of relatively small particles. EDX spectra for the samples can be found in Supplementary Materials (Figures S3 and S4).

**Figure 5:**
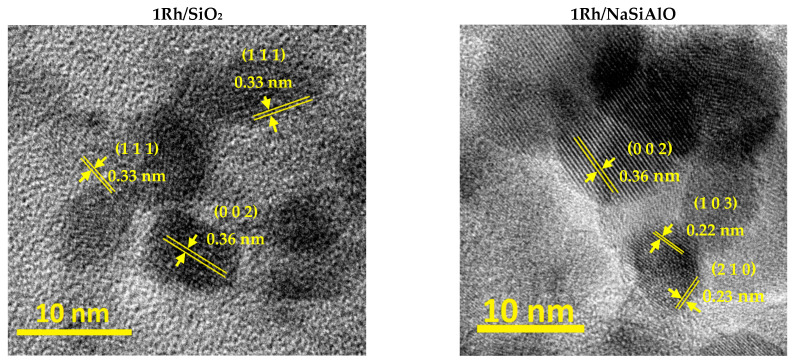
TEM images of the initially calcined in air catalysts with Si-containing supports. The crystal planes are indicated in the pictures.

The measured interplanar spacings for the sample 1Rh/SiO_2_ were 0.36, and 0.33 nm for the areas of crystallinity on the TEM picture (Figure 5). These interplanar spacings correspond to a Rh_2_O_3_ phase, the ICDD card number [24-924]. The revealed interplanar spacings for the sample 1Rh/NaSiAlO were 0.36, 0.22, and 0.23 nm for the different areas on the TEM image. These interplanar distances also correspond to the Rh_2_O_3_ phase. The estimation of the phase composition was performed because of the impossibility to determine it from XRD data.

The two other catalysts (prepared from the supports without silica) do not demonstrate any crystal planes. Perhaps, it results from the support nature and the specificity of the support-metal sites interaction in terms of the effect of Broensted acidity. Nevertheless, the catalysts were used only after reduction in an H_2_ flow, which is why the rhodium (III) oxide phase is transformed into the metal sate.

**Figure 6:**
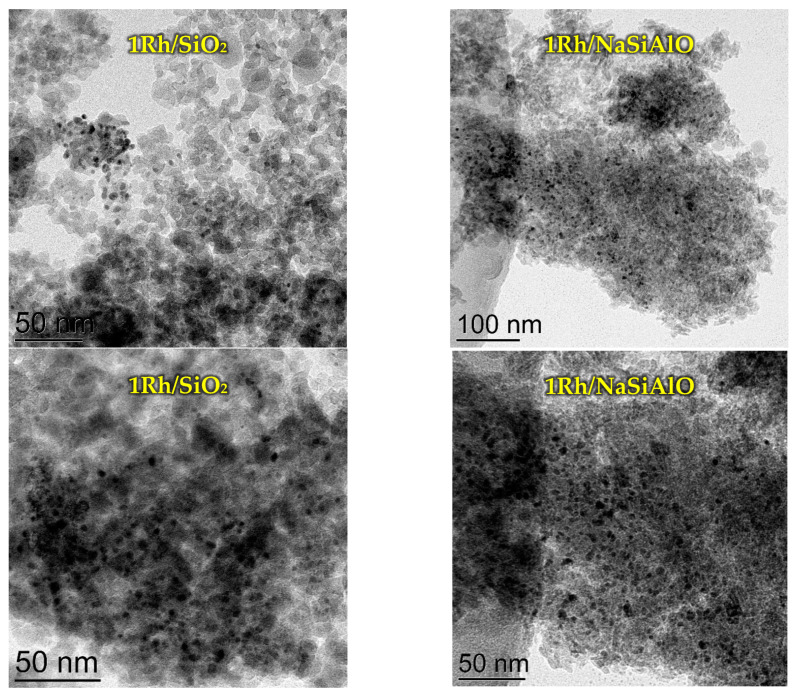
TEM images of the reduced H_2_ catalysts with Si-containing supports.

The catalysts with silicon-containing supports were additionally investigated after reduction with H_2_ (Figure 6). It can be seen that the samples contain small Rh-containing particles (dark points in the pictures).

**Figure 7:**
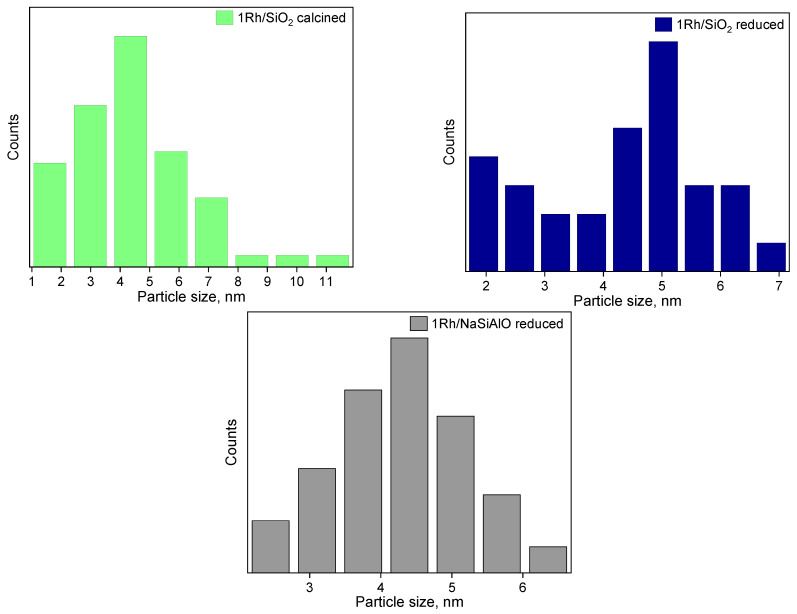
Rh particle distribution by size in the case of initially calcined in air and reduced catalysts.

**Figure 8:**
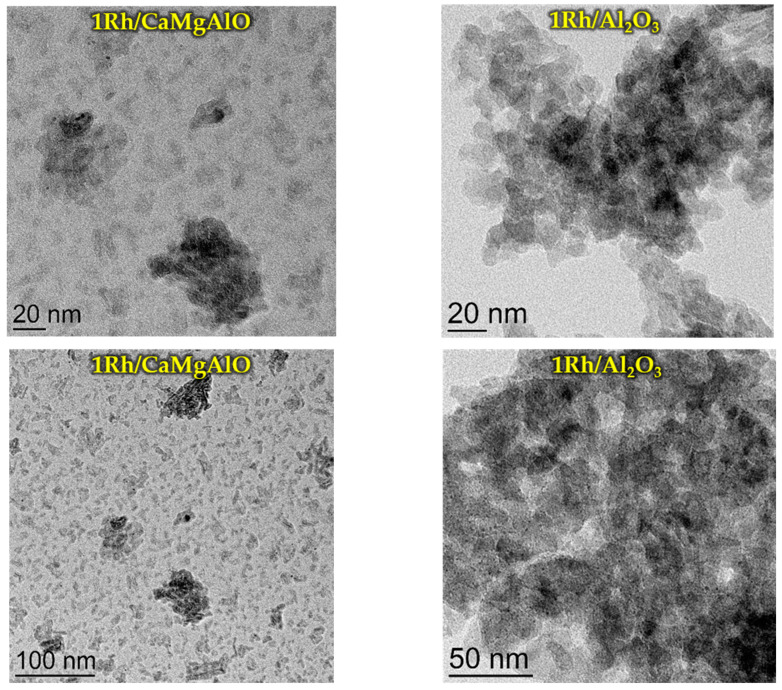
TEM images of the reduced H_2_ catalysts with Al-containing supports.

The distribution of Rh particles by size (Figure 7) differs from the sample 1Rh/SiO_2_ initially calcined in air to a reduced one. It can be seen that the distribution became bimodal, a maximum at 4 nm disappeared and two smaller maxima appear at 2 and 5 nm (seemingly because of reduction). Moreover, in the case of the sample 1Rh/NaSiAlO, it became possible to reveal the rhodium nanoparticles on the surface only after the reduction. Figure 8 provides TEM images of the reduced samples 1Rh/CaMgAlO and 1Rh/Al_2_O_3_. It can be noticed that, after the reduction, the nanoparticles of Rh appeared, but the numbers of observable particles for these samples are less than for the samples with silica-based supports. Generally, it can be seen that all the samples being reduced demonstrate the nanoparticles of Rh, while only silica-based ones do in the case of calcined in air samples.

### 3.5. UV-Vis Diffuse Reflectance Spectroscopy

The UV-Vis DRS results can be found in Supplementary Materials (Figure S5) with the interpretation of Rh^3+^ bands [27,28,29].

### 3.6. DRIFT Spectroscopy

All the supports used for the catalyst preparation were investigated by DRIFT spectroscopy.

**Figure 9:**
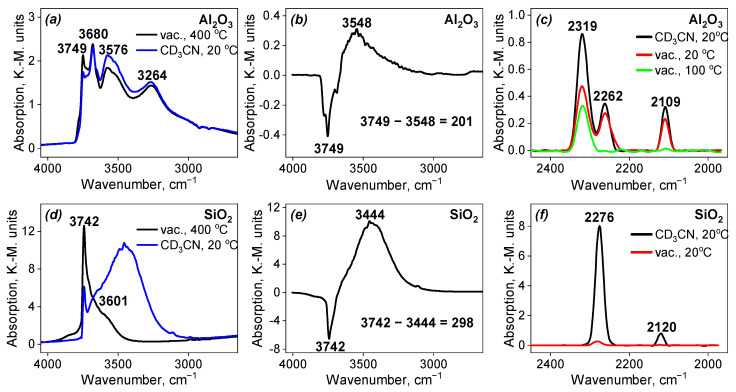
Spectra of single-component supports: (**a**,**d**) DRIFT-OH after evacuation and after adsorption of CD_3_CN; (**b**,**e**) difference of the DRIFT-OH spectra before and after adsorption of CD_3_CN; (**c**,**f**) DRIFT-CD_3_CN adsorption-desorption spectra of the support.

The results for the single-component supports, i.e., Al_2_O_3_ and SiO_2_, are presented in Figure 9, Table 2 and Table 3. In the case of Al_2_O_3_, it can be seen that the intensity of each of the four observed bands is higher for the sample after the adsorption of CD_3_CN compared to the sample after evacuation under heating. In the case of SiO_2_, the intensity of the band in the spectrum of the evacuated sample decreased while a new wideband appeared. The assignment of all the bands is given in Table 2 and Table 3. The difference in the spectra before and after CD_3_CN adsorption gives the values of the shifts of 201 cm^−1^ and 298 cm^−1^, respectively. It indicates that the sample of Al_2_O_3_ contains weak Broensted acid sites (BAS) on the surface, while SiO_2_ contains moderate ones. Both these observations are proven by the shifts of the CN stretching vibration band. The alumina support also contains Lewis acid sites (LAS) on the surface (Table 3). The Al_2_O_3_ support demonstrates a disappearance of the band from BAS, while LAS bands are present in the spectrum up to 100 °C under evacuation. At the same time, the band from BAS disappears in the spectrum of SiO_2_ already at room temperature during evacuation. The band in the region of 2110–2120 cm^−1^ corresponds to the bending vibrations of CD_3_ groups.

**Figure 10:**
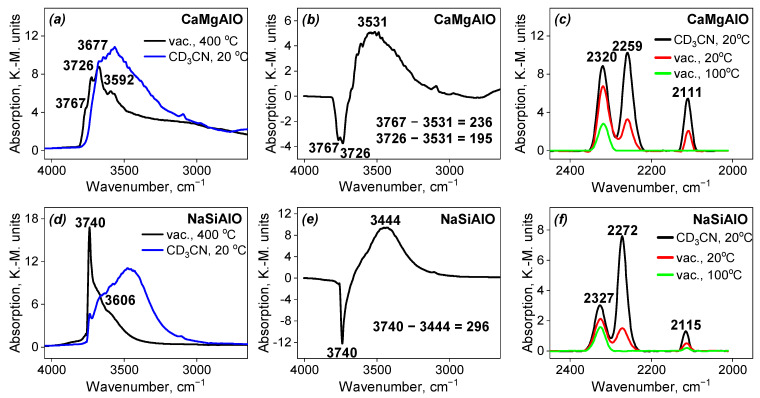
Spectra of mixed-component supports: (**a**,**d**) DRIFT-OH after evacuation and after adsorption of CD_3_CN; (**b**,**e**) difference of the DRIFT-OH spectra before and after adsorption of CD_3_CN; (**c**,**f**) DRIFT-CD3CN adsorption-desorption spectra of the support.

The results for the mixed-component supports, i.e., CaMgAlO and NaSiAlO, are presented in Figure 10, Table 2 and Table 3. For both these supports, the appearance of a wide band upon CD_3_CN adsorption can be seen. The assignment of all the bands is given in Table 2 and Table 3. The difference of the spectra before and after CD_3_CN adsorption gives the values of the shifts of 236 and 195 cm^−1^ for the sample CaMgAlO and 296 cm^−1^ in the case of NaSiAlO. It indicates that the sample CaMgAlO contains weak BAS on the surface, while NaSiAlO contains BAS of moderate strength, which can be explained by the presence of silicon in the composition. Both these observations are proven by the shifts of the C≡N stretching vibration band. Both these samples demonstrate LAS on the surface, which remains in the spectra after thermal desorption up to 100 °C. BAS bands disappeared first for both samples.

The strength of Broensted acid sites increases in the order: SiO_2_ ≅ NaSiAlO > CaMgAlO > Al_2_O_3_ (Table 2). The strength of Lewis acid sites is about the same for all the samples, excluding SiO_2_, which does not demonstrate any Lewis acidity.

### 3.7. XPS

The XPS investigation was performed for all the catalysts (Table 4) and the state of rhodium on the surface was examined (Table 5). The found compositions are close to the calculated ones. The weight percentage of Rh atoms on the surface differs from the value expected by design: it might be the result of an inhomogeneous distribution of rhodium atoms assuming the local nature of the analysis. Nevertheless, the obtained values are close to the desirable 1 wt%. The error of the method let us perform only a qualitative estimation of the Rh content on the surface.

### 3.8. TPR-H_2_

**Figure 11:**
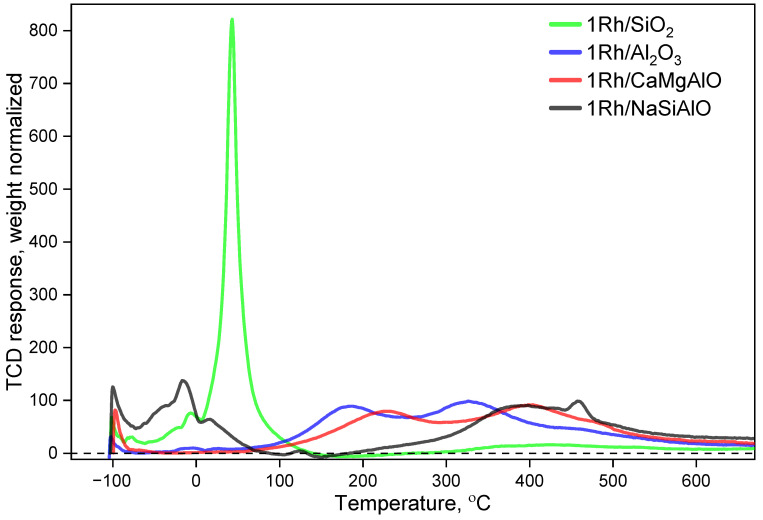
TPR-H_2_ profiles for the rhodium-loaded catalysts.

The results of TPR-H_2_ examinations are shown in Figure 11 and Table 5. All the rhodium-loaded samples demonstrate peaks of hydrogen consumption in the region of low temperatures. By the way, the additional peaks at higher temperatures but still below the zero line can be seen only for the silica-containing samples. It can be attributed to the interactions of rhodium (probably, oxide) particles with the support, particularly, with the silica species. Furthermore, such samples demonstrate high-temperature peaks at significantly higher temperatures than the peaks of alumina-based samples (the shift is more than 200 °C). Being compared, the silica-containing samples differ in that they have peaks of hydrogen desorption of low intensities at different temperatures: the relatively wide peak of hydrogen desorption for the sample 1Rh/SiO_2_ is found at the temperature of 175 °C, while the sample 1Rh/NaSiAlO demonstrates two small peaks at 105 and 150 °C. At the same time, the maximal intensity peak is located at about 45 °C for the sample 1Rh/SiO_2_, while in the case of the sample 1Rh/NaSiAlO, it is found in the region of about 400 °C. Such a difference in reducibility may be caused by the difference in the composition of the sample: the silicon oxide species seem to provoke the reduction of rhodium oxide particles at much lower temperatures: it obviously also resulted from the smaller size of particles in the case of the SiO_2_-based catalyst, which can be attributed to the specific interactions between the active phase and the support. Additionally, the narrowness of the peak of hydrogen consumption at about 45 °C allowed us to propose the relative uniformity of the rhodium-oxide particles by their size.

The most low-temperature peaks are related to argon desorption. The peaks at temperatures above –60 °C can be attributed to hydrogen consumption. Additionally, we can see small peaks of hydrogen desorption only for the silica-containing samples over the region of about 200 °C. In the case of the sample 1Rh/SiO_2_, such a desorption peak constitutes about 1.5% of the overall hydrogen consumption, while in the case of the sample 1Rh/NaSiAlO—only 0.7%. In the case of the other samples, which do not contain any silicon, no desorption peaks can be seen at all. So, these peaks can be attributed to silicon-containing samples only.

The overall hydrogen consumption (H_2_/Rh) decreases in the following order: 1Rh/NaSiAlO > 1Rh/CaMgAlO ≈ 1Rh/Al_2_O_3_ > 1Rh/SiO_2_. The hydrogen consumption at temperatures below 300 °C along with Rh reduction could also correspond to the chemical adsorption of hydrogen on the surface of the samples and the surface rehydroxylation by hydrogen activated over Rh^0^ species (from –Si–O–Si– groups). At temperatures above 300 °C the reduction of impurities that may be present in the industrially prepared supports can be also proposed. Nevertheless, the amounts of these impurities are not too large, otherwise, they would be observed by EDX or XPS. In the case of the MgO-containing support, the additional possible way is the reduction of Rh_2_MgO_4_ (reduced at 425–625 °C according to the literature data [49,50,51,52]). In the case of 1Rh/Al_2_O_3_, the two observed peaks are in accordance with the literature data [53], but the temperature range in our case is shifted to higher temperatures. It may be a result of the higher particle size of rhodia. Generally, two groups of peaks are commonly distinguished: so-called “easy-to-reduce” peaks and “difficult-to-reduce” peaks [54,55,56,57].

### 3.9. The Catalytic Tests

**Figure 12:**
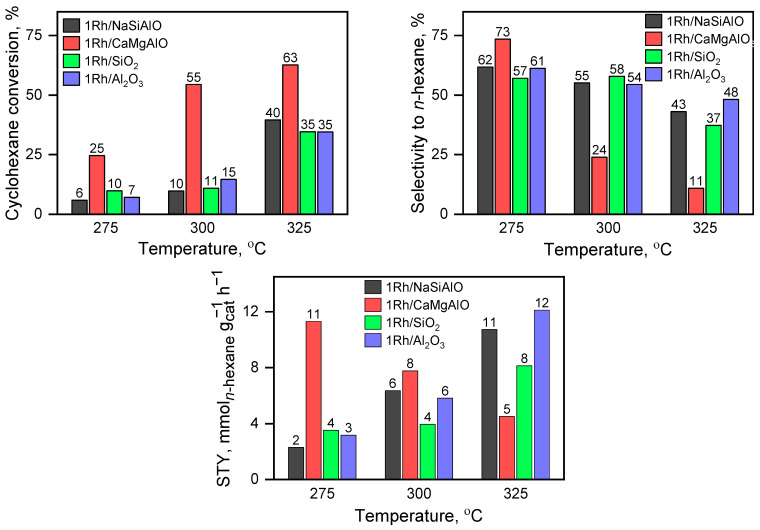
The results of the catalytic tests in cyclohexane ring opening: (**left**) cyclohexane conversion; (**right**) selectivity to *n*-hexane; (**bottom**) STY of *n*-hexane.

All the prepared catalysts were tested in a cyclohexane ring opening, the results of the examinations are presented in Figure 12. All the catalysts demonstrate non-zero activities in terms of cyclohexane conversion. The trend toward increasing conversion of cyclohexane can be seen for all the samples.

The sample 1Rh/CaMgAlO shows the maximal selectivity to n-hexane at 275 °C, but it decreases significantly when the temperature increases: at the temperatures of 300 and 325 °C, the selectivity took the minimal value among all the investigated catalysts. This sample demonstrates the maximal conversion of cyclohexane at each applied temperature. It results in the maximal space-time yield of n-hexane among the catalysts at 275 and 300 °C, but at 325 °C this catalyst is the least productive. It is noticeable that the STY of n-hexane obtained using the catalyst 1Rh/CaMgAlO is about the same as for the other most productive samples (1Rh/NaSiAlO and 1Rh/Al_2_O_3_), but the temperature in the case of the sample 1Rh/CaMgAlO is 50 °C lower. The STY of n-hexane increases with increasing temperature for all the samples, except for 1Rh/CaMgAlO: in the case of this sample, the dependence is reversed. The detailed catalytic data can be found in Supplementary Materials (Table S2).

## 4. Discussion and Conclusions

Since the samples have a similar pore size distribution, except for the sample SiO_2_, which has the lowest activity and selectivity to desirable products, so, the factor of the surface area and pore size distribution can be excluded from the analysis in terms of the effects on the catalytic activity and selectivity to *n*-hexane.

Phase compositions can be estimated only in terms of the intensity of the Al_2_O_3_ phase reflexes, which decreases as follows: 1Rh/Al_2_O_3_ > 1Rh/CaMgAlO > 1Rh/NaSiAlO. It seems to be caused by the presence of other components. The alumina phase crystallinity also should not affect the catalytic behavior dramatically. We can propose that the crystallinity of the active phase of Rh could be responsible for the difference in the catalytic activity (TPR-H_2_ experiment), but its crystallinity cannot be estimated from the data obtained.

One can conclude from the SEM and EDX data that only the sample 1Rh/NaSiAlO demonstrates a strongly inhomogeneous rhodium distribution on the surface, and at the same time the catalytic activity of this sample is moderate, while STY of *n*-hexane is moderate too, except for the temperature of 325 °C: in this case, the STY of n-hexane is close to the maximal value. Possibly, agglomeration of Rh species on the surface can be the cause of the decrease in the cyclohexane conversion, while highly dispersed Rh particles favor more effective performance.

It can be seen from the data obtained by the TEM technique that the three most active samples have similar rhodia particle sizes, but SiO_2_ has much smaller ones, then follows the sample 1Rh/NaSiAlO. Nevertheless, the conversion of cyclohexane for these samples does not differ dramatically. It is also in agreement with TPR-H_2_ data, which revealed that the samples 1Rh/SiO_2_ and 1Rh/Al_2_O_3_ have the lowest reducibility. The distinct peak for the sample 1Rh/SiO_2_ may also indicate the higher homogeneity of rhodium oxide particles by their size. Thus, one can see the trend in that the samples’ reducibility decreases simultaneously with the decrease in the selectivity to n-hexane. This may be explained as follows: the species that are most active in the reaction can be produced from such reducible particles. Nevertheless, Rh nanoparticles were found in each of the reduced catalysts, but their observed amounts were different: the most abundant Rh nanoparticles were found in the samples with silica-based supports and much less—in alumina-based ones.

The results of DRIFT spectroscopy are consistent with the observations above. From the viewpoint of the catalytic activity, noticeable is the fact that the sample with the support CaMgAlO is slightly more acid in terms of BAS and the most productive to *n*-hexane at 275 and 300 °C, but not at higher temperatures because of the lower conversion of cyclohexane. This allowed us to propose that the optimal Bronsted acidity is attained in this sample. At higher temperatures, the BAS may partly disappear. It is also consistent with the literature data [31].

So, the optimal catalyst 1Rh/CaMgAlO should contain BAS with moderate strength. In this case, the attained selectivity to *n*-hexane is about 75% at the conversion of cyclohexane of 25%.

## Figures and Tables

**Table 1:** The textural properties of the supports of the catalysts derived by the N_2_ low-temperature adsorption-desorption technique.

Sample	S_BET_, m^2^g^−1^	V_total_, cm^3^g^−1^	V_meso_, cm^3^g^−1^	V_micro_, cm^3^g^−1^	D_max_, nm
SiO_2_	239	0.92	0.92	0.005	12.9
Al_2_O_3_	96	0.27	0.27	0.016	9.8
CaMgAlO	245	0.58	0.57	0.011	7.7
NaSiAlO	410	0.58	0.57	0.015	1.6, 6.4

**Table 2:** The assignment of DRIFT spectroscopy bands in the region of OH groups.

Sample	Frequency, cm^−1^	Bond Type	Literature
Al_2_O_3_	3749	linear isolated Al-OH	[30,31,32,33,34,35,36,37]
	3680	bridged OH	
	3576	H-bonded OH	
	3264	contaminants	
SiO_2_	3742	isolated Si-OH	[25,35,36,38,39,40,41,42]
	3601 (shoulder)	bridged OH (or SiOH nests)	
CaMgAlO	3726	linear isolated Al-OH	[35,37,43,44,45]
	3767 (shoulder)	linear isolated Mg-OH	
	3677	bridged OH *	[46]
	3592	H-bonded OH	[30,31,32,33,34,35,36,37]
NaSiAlO	3740	isolated Si-OH	[25,35,36,38,39,40,41,42]
	3660	bridged OH	

* Probably the bridge is formed between Ca and Al as it was reported for Ca-BEA [46].

**Table 3:** The shifts of stretching vibrations of CN groups upon interaction with CD_3_CN (strong base) and the blue shift of stretching ν C≡N vibrations compared to the gas phase frequency (2253 cm^−1^) [35,37,47,48].

Sample	Frequency, cm^−1^	Blue Shift of CN Stretching Vibration, cm^−1^	Corresponding Sites *
Al_2_O_3_	2319	66	LAS
	2262	9	bridged hydroxyl groups
SiO_2_	2276	23	moderate BAS
CaMgAlO	2320	67	LAS
	2259	6	bridged hydroxyl groups
NaSiAlO	2327	64	LAS
	2272	19	moderate BAS

* BAS—Broensted acid sites. LAS—Lewis acid sites.

**Table 4:** The atomic concentrations of the elements on the surface of non-reduced rhodium-loaded catalysts by XPS.

Sample	O, (±1.0) at.%	Al, (±0.3) at.%	Si, (±0.3) at.%	Rh, (±0.1) at.%	Rh, wt%
SiO_2_	65.8	—	30.9	0.1	0.5
NaSiAlO	63.8	17.6	13.2	0.1	0.5
CaMgAlO	60.0	32.2	—	0.3	1.7
Al_2_O_3_	58.1	33.5	—	0.2	1.1

**Table 5:** TPR-H_2_ results for non-reduced catalysts.

Sample	T_max_ for Main Peaks, °C	H_2_/Rh Ratio (mol.)
1Rh/SiO_2_	−7, +43, +425	1.62
1Rh/Al_2_O_3_	+185, +330	1.96
1Rh/CaMgAlO	+230, +400	2.03
1Rh/NaSiAlO	−15, +390, +460	2.38

## Data Availability

The data are available from the authors upon request.

## Supplementary Materials

The following supporting information can be downloaded at: https://www.mdpi.com/article/10.3390/nano13050936/s1, Additional materials on the physicochemical characterization of the samples and the catalytic data: Figure S1. TPR-H_2_ profile of the sample 9%Rh/SiO_2_(Acros). Figure S2. Isotherms of N_2_ low-temperature adsorption-desorption. Figure S3. EDX spectrum for the sample 1Rh/NaSiAlO (from TEM characterization). Figure S4. EDX spectrum for the sample 1Rh/SiO_2_ (from TEM characterization). Figure S5. UV-visible diffuse reflectance spectra of rhodium-loaded catalysts after calcination under air atmosphere. The dashed lines demonstrate the positions of allowed transitions of supported Rh^3+^. Table S1. The EDX results for non-reduced catalysts: (a) 1Rh/NaSiAlO, (b) 1Rh/CaMgAlO, (c) 1Rh/SiO_2_, (d) 1Rh/Al_2_O_3_. Table S2. The results of catalytic tests in the cyclohexane ring opening reaction under the pressure of 40 atm. The feed mixture consisted of 0.0170 mL of cyclohexane (liquid, further vaporized) per minute and an H_2_ (gas) flow of 50 mL per minute.
