# Supplementary material for: Rhodium-Based Catalysts: An Impact of the Support Nature on the Catalytic Cyclohexane Ring Opening
Source: Nanomaterials (Basel). 2023 Mar 4;13(5):936. doi: 10.3390/nano13050936 (PMC10005695; doi:10.3390/nano13050936)
Supplement: Supplementary file 1 [file nanomaterials-13-00936-s001.zip › nanomaterials-2226160-supplementary.pdf]

Supporting information for the article

Rhodium-Based Catalysts: An Impact of the Support Nature on the Catalytic Cyclohexane Ring Opening

Kristina E. Kartavova [1,2], Mikhail Yu. Mashkin [1,2,3], Mikhail Yu. Kostin [1], Elena D. Finashina [3], Konstantin B. Kalmykov [1], Gennady I. Kapustin [3], Petr V. Pribytkov [1,3], Olga P. Tkachenko [3], Igor V. Mishin [3], Leonid M. Kustov [1,2,3,*] and Alexander L. Kustov [1,2,3]

[1] Department of Chemistry, Lomonosov Moscow State University, 119991 Moscow, Russia

[2] Institute of Ecotechnologies, National University of Science and Technology "MISiS", 119049 Moscow, Russia

[3] N.D. Zelinsky Institute of Organic Chemistry RAS, 119991 Moscow, Russia

* Correspondence: lmkustov@mail.ru

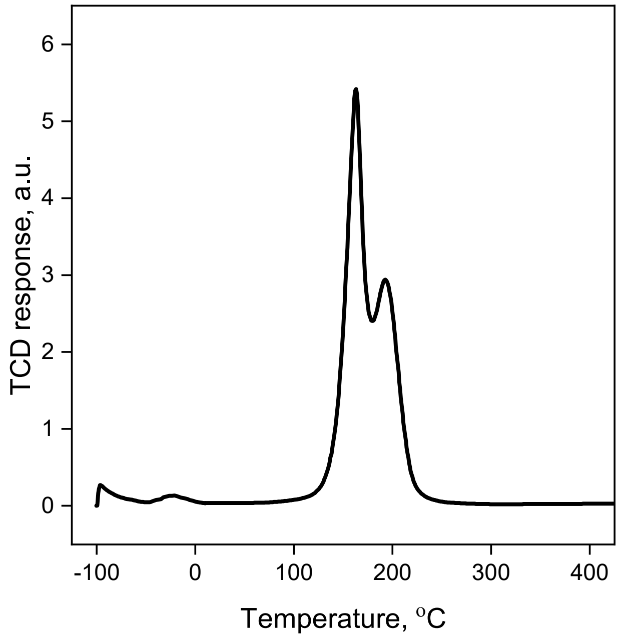

Figure S1. TPR-H_2 profile of the sample 9%Rh/SiO_2(Acros).

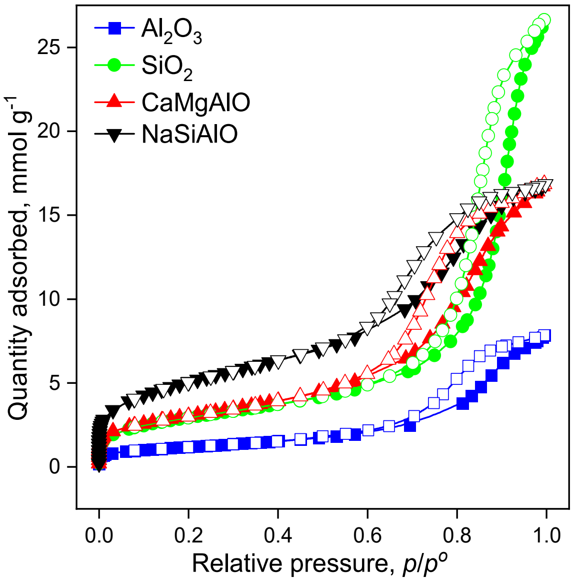

Figure S2. Isotherms of N_2 low-temperature adsorption-desorption.

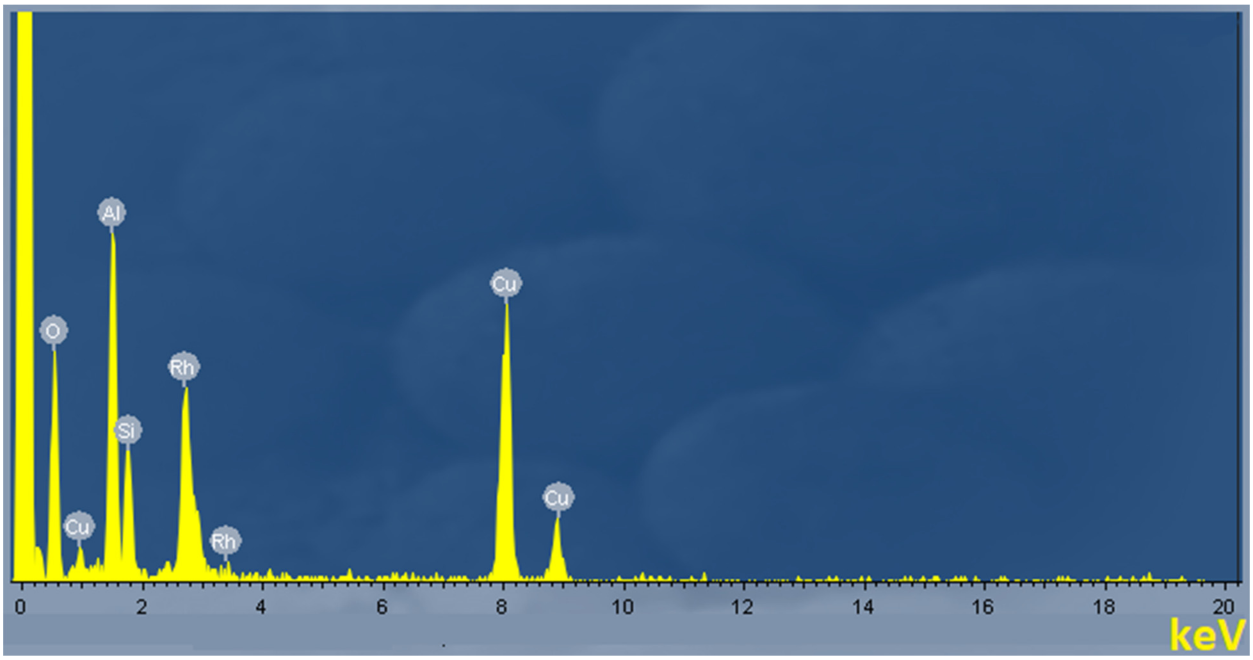

Figure S3. EDX spectrum for the sample 1Rh/NaSiAlO (from TEM characterization).

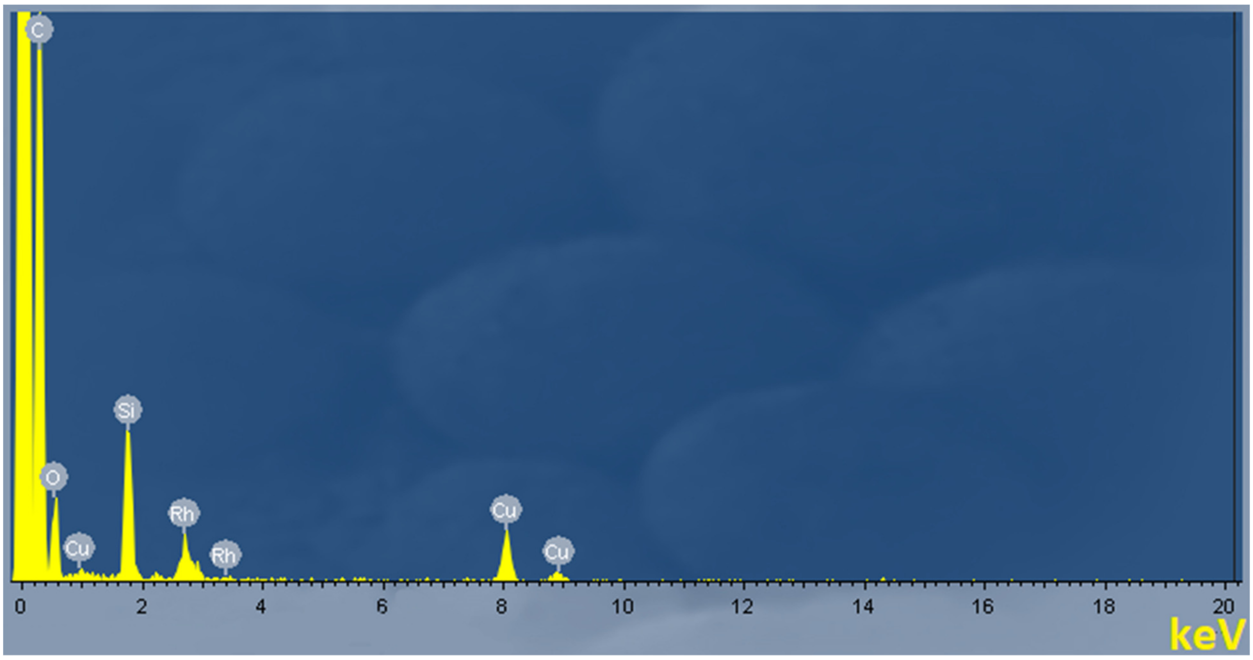

Figure S4. EDX spectrum for the sample 1Rh/SiO_2 (from TEM characterization).

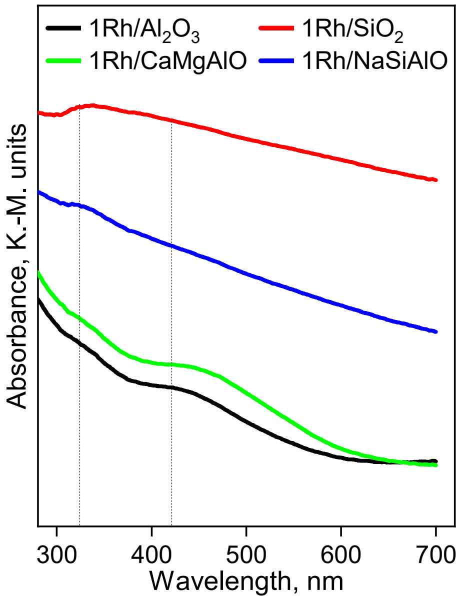

Figure S5. UV-visible diffuse reflectance spectra of rhodium-loaded catalysts after calcination under air atmosphere. The dashed lines demonstrate the positions of allowed transitions of supported Rh^{3+} [24–26].

The optical properties of the samples were examined by UV-visible diffuse reflectance spectroscopy. The difference between the samples is clear: the samples demonstrate a decrease in the absorbance in the following row: 1Rh/SiO_2 > 1Rh/NaSiAlO > 1Rh/CaMgAlO > 1Rh/Al_2O_3.

Two bands with maxima at about 410 and 320 nm correspond to Rh^{3+} species on the alumina support according to the literature [24–26]. Additionally, a charge transfer band can be seen at about 250 nm.

Table S1. The EDX results for non-reduced catalysts: (a) 1Rh/NaSiAlO, (b) 1Rh/CaMgAlO, (c) 1Rh/SiO_2, (d) 1Rh/Al_2O_3.

(a) 1Rh/NaSiAlO

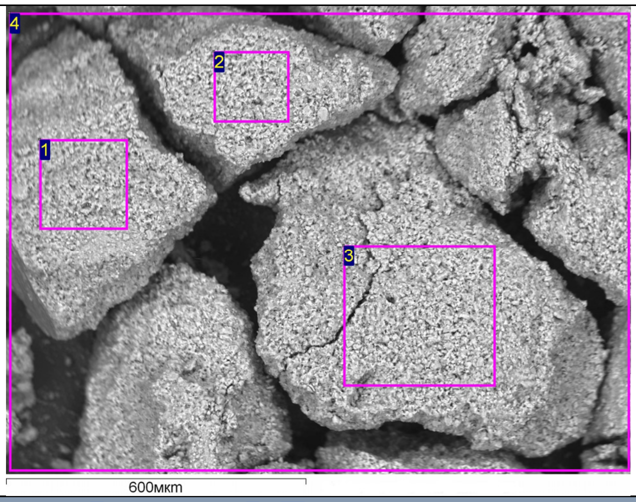

Entry	O	Na	Al	Si
1	52.0	0.2	13.9	33.9
2	51.6	0.2	13.9	34.3
3	53.5	0.0	13.0	33.5
4	55.1	0.2	12.9	31.8

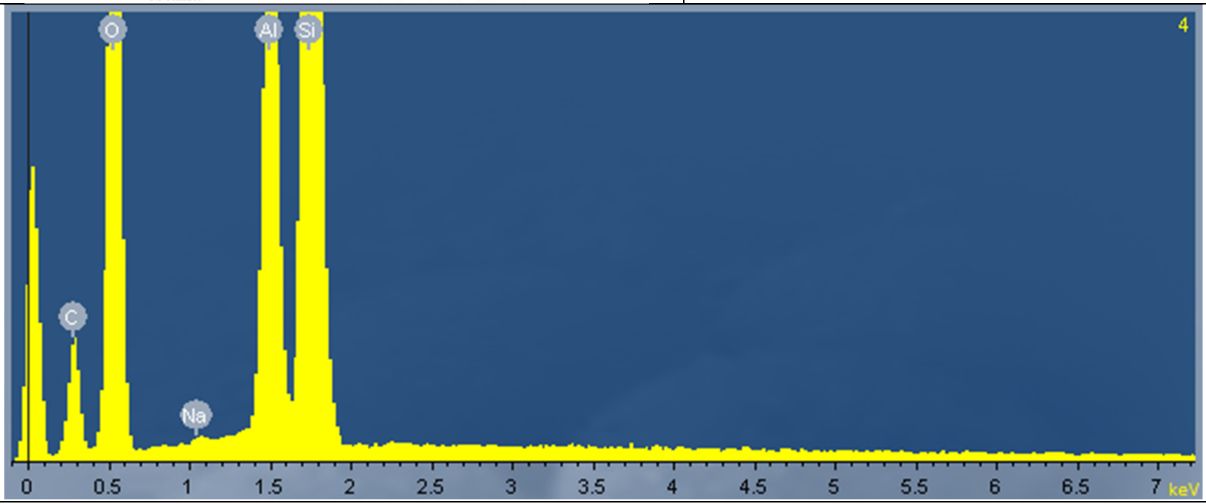

(b) 1Rh/CaMgAlO

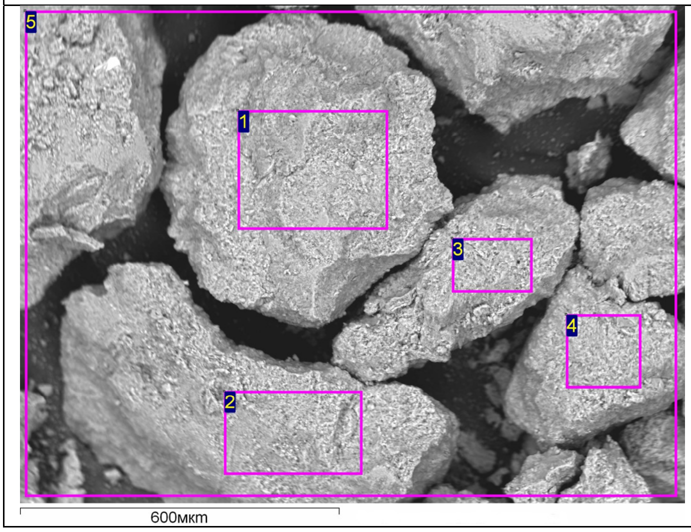

Entry	O	Al
1	52.0	48.0
2	49.2	50.8
3	51.3	48.7
4	45.3	54.8
5	52.3	47.7

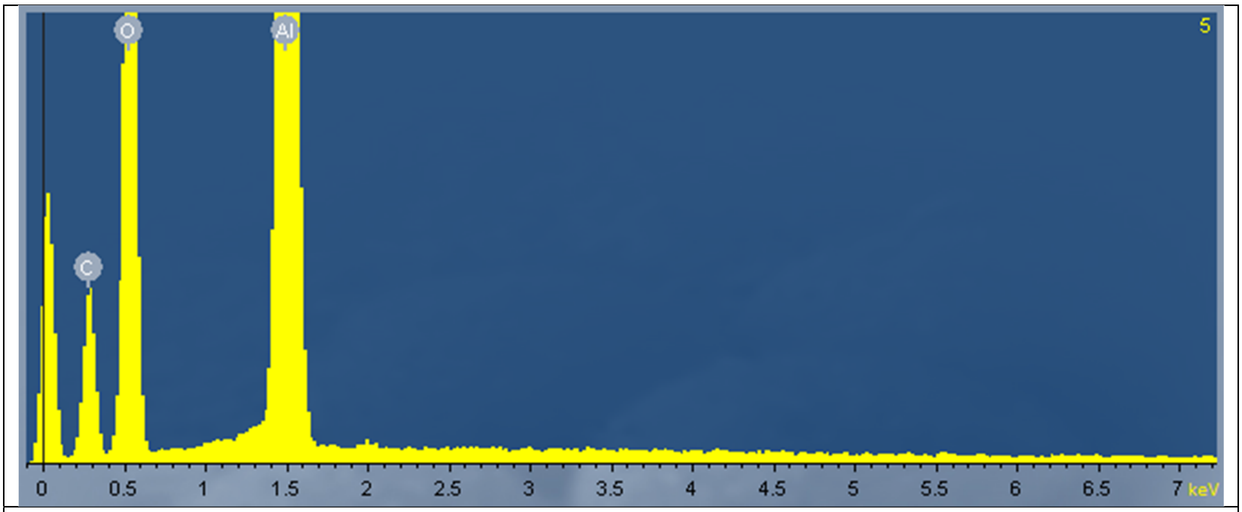

(c) 1Rh/SiO_2

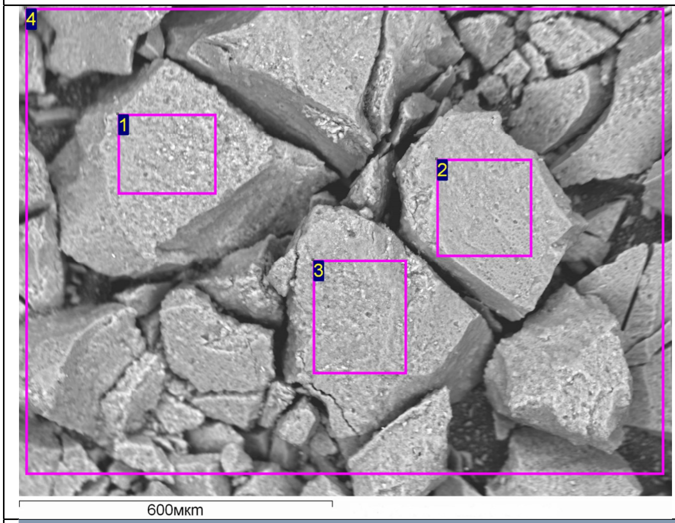

Entry	O	Si
1	51.3	48.7
2	55.2	44.8
3	51.6	48.4
4	54.1	45.9

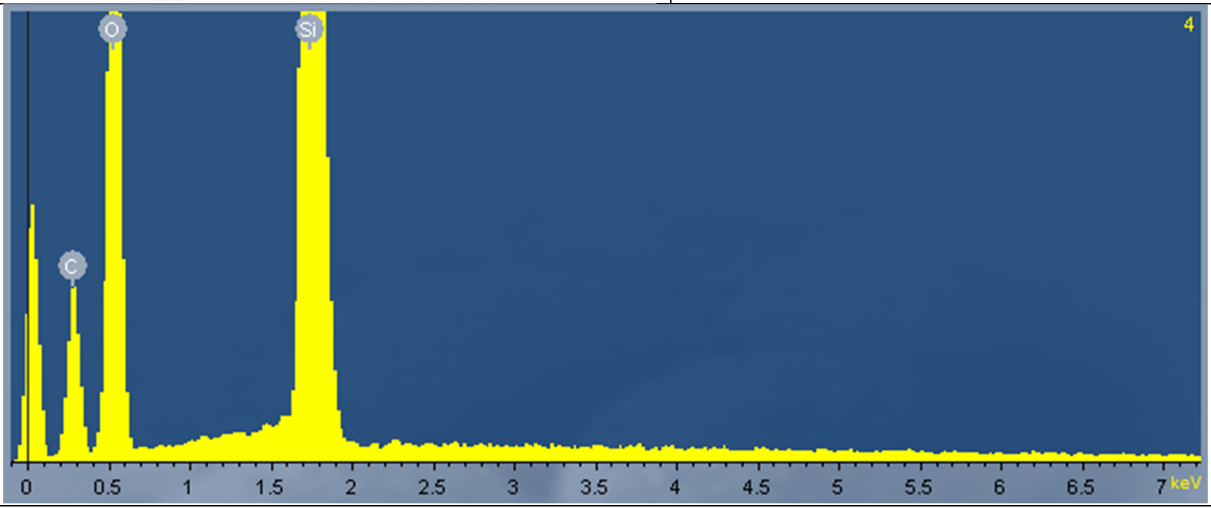

(d) 1Rh/Al_2O_3

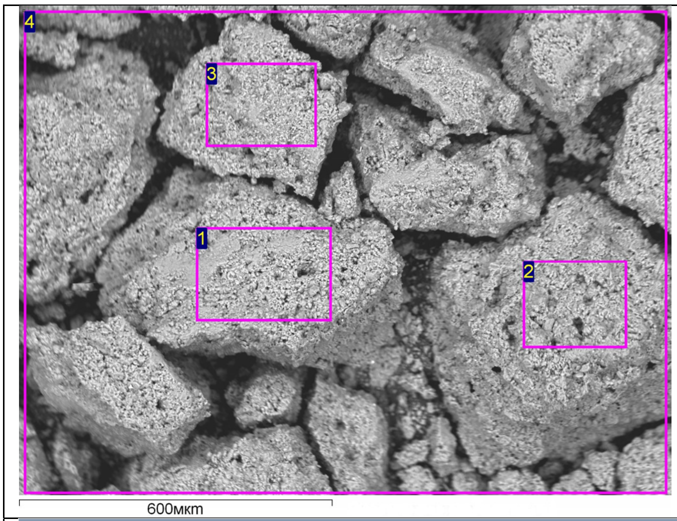

Entry	O	Al
1	48.7	51.4
2	50.2	49.8
3	48.4	51.6
4	51.4	48.6

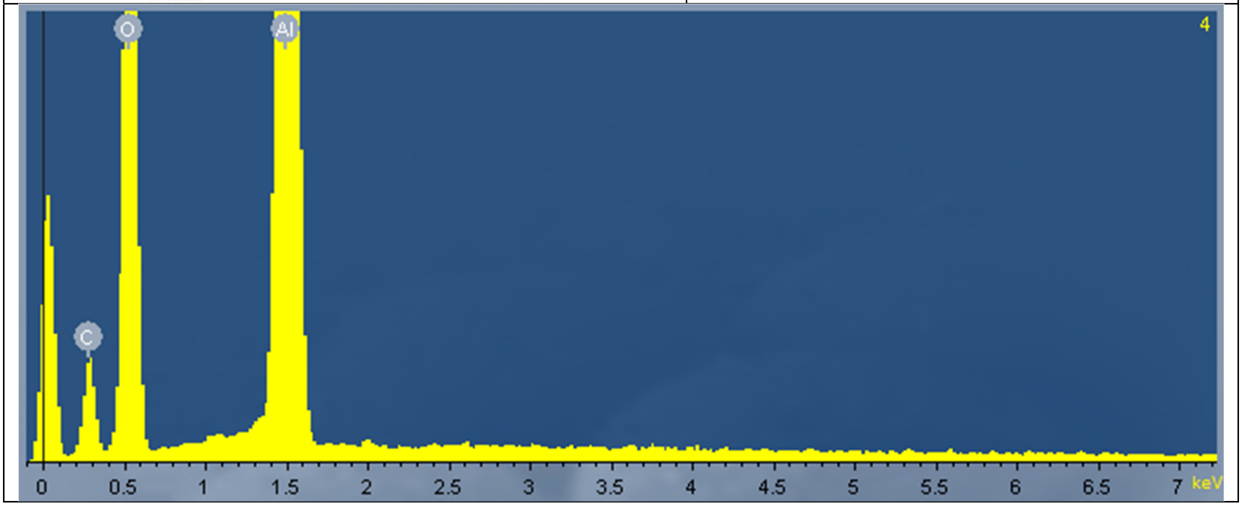

Table S2. The results of catalytic tests in the cyclohexane ring opening reaction under the pressure of 40 atm. The feed mixture consisted of 0.0170 ml of cyclohexane (liquid, further vaporized) per minute and H_2 (gas) flow of 50 ml per minute.

**Sample**	**T, °C**	**Conversion of cyclo-C6, %**	**Selectivity to *n*-C6, %**	**Selectivity to C1–C5, %**
1%Rh/Al_2O_3	275	7.1	61.2	38.5
	300	14.7	54.5	45.5
	325	34.6	48.2	51.3
1%Rh/CaMgAlO	275	24.6	73.5	25.9
	300	51.5	24.0	75.1
	325	62.7	10.9	88.3
1%Rh/SiO_2	275	9.8	57.1	41.6
	300	10.9	57.9	40.9
	325	34.7	37.3	61.6
1%Rh/NaSiAlO	275	5.9	61.8	36.5
	300	9.7	55.2	42.8
	325	39.6	43.1	54.2
